# Book Reviews

**Published:** 1900-08

**Authors:** 


### Book Reviews.

DISEASES OF THE STOMACH, THEIR SPECIAL PATHOLOGY, DIAGNOSIS AND TREATMENT. With Sections on Anatomy, Physiology, Chemical and Microscopical Examination of Stomach Contents, Dietetics, Surgery of the Stomach, etc. By JOHN C. HEMMETER, M. D., Professor in the Medical Department of the University of Maryland, Baltimore. With many original illustrations, a number of which are in colors. Second edition, enlarged and revised. Octavo, 898 pages. Philadelphia: P. Blakiston's Son & Co., 1012 Walnut Street. 1900. [Price, $6.00 net, cloth.]

About two years ago (May, 1898,) we noticed the first edition of this book in extenso. It was the first American treatise to deal with the subject exhaustively, or with sufficient comprehensiveness to entitle the work to be classed in the text-book group.

This new edition follows the general plan of the first in its make-up, as well as in the method of its treatment of the several branches of the subject matter considered in the book. After dealing with the anatomy and physiology of the digestive organs, the therapy and materia medica of digestive diseases, and the surgical treatment of organic gastric disorders, Hemmeter then takes up special diseases of the stomach in a section of the work, called the gastric clinic. This comprises about one-half the bulk of the volume, as it did in the first instance. It deals with gastritis, both acute and chronic, ulcer of the stomach, malignant tumors, tuberculosis and syphilis of the stomach, benign tumors, motor insufficiency, hemorrhage, enteroptosis, neurosis of the stomach, achylia gastrica, nervous dyspepsia, etc., etc.

This is a practical part of the work and in many instances the author is both original and ingenious in his methods of diagnosis and treatment. The importance of an intimate knowledge of diseases of the stomach is a growing necessity, and every contributor to the sum of this knowledge should have a patient ear and an inquiring mind for an auditor in the person of the general practitioner of medicine. It is to him that this work appeals in particular, yet the abdominal surgeon may not neglect it, for it contains much with which he must become familiar before he can call himself well-equipped to do the surgery of the stomach.

This second edition of Hemmeter is practically a new treatise, since so much of it has been rewritten, and all of it has practically been reconstructed. Moreover, much new material has been added, among which the more important articles are: hypertrophic stenosis of the pylorus, obstruction of the orifices, use and abuse of rest in the treatment of digestive diseases, and parts of chapters on other subjects.

New illustrations and plates, too, have been added in order to carry it forward to the immediate present in its teachings. Progress has been so rapid in this topic, and is continuing to move ahead with so much speed, that one must needs watch the literature of it carefully. Divested of the necessary sentiments attached to the prefaces and the forepages expressed in fable and poetry, we regard the book as of the best on the stomach and its diseases that have issued from an American press and by an American author. The indices of authors and subjects are most complete—a very important part of a good book.

ANNUAL AND ANALYTICAL CYCLOPEDIA OF PRACTICAL MEDICINE. By CHARLES E. DEM. SAJOUS, M. D., and 100 associate editors. Assisted by corresponding editors, collaborators and correspondents. Illustrated with chromo-lithographs, engravings and maps. Volume V. Methyl-Blue to Rabies. Philadelphia, New York and Chicago: F. A. Davis Co. 1900.

This volume adds to the previous reputation of Sajous's Annual in its present form, and is one of the most important of the series. It embraces nearly all the specialties, containing as it does articles in otology, laryngology, ophthalmology, pediatrics, obstetrics, therapeutics and others, as well as sections classed under the head of general medicine and surgery.

The editor invites special attention to the articles on nursing and artificial feeding by Holt and Fètra; disorders of pregnancy, by Currier; abnormal parturition, by Grandin and Marx; pleurisy, by McPhedran; catarrhal pneumonia, by Solis-Cohen, and lobar pneumonia, by Ashton. In the section on nursing and artificial feeding, we are surprised to find no reference to the service Ernest Wende has rendered in the suppression of the long-tube nursing bottle, although under the sub-head, "Bottles and Nipples," the importance of attention to the feeding receptacle is referred to and the long rubber tube is condemned, quoting Rothschild on the subject. As a matter of fact the glass tube is equally dangerous as has been repeatedly pointed out by Wende, and demonstrated by photo-micrographs and otherwise. Indeed, an ordinance is in force in Buffalo prohibiting the sale of the long-tube nursing bottle, the only city within our knowledge where such action has been taken. The result has been a diminution of the infantile death-rates of more than 50 per cent.

Of special interest in Buffalo and vicinity is the subject of rabies, in view of the epidemic that has recently existed. The article on rabies, by Bondurant, is a résumé of our present knowledge of the subject. It is surprising how sparse the literature of rabies is, but it is probable that it will soon increase as Wende is also devoting much attention to this subject having recently presented some results of his studies to the American Medical Association at its Atlantic City meeting.

Taken together the subjects presented in this volume are unsurpassed in their interest and importance by any of the previous numbers of the series. It is one of the most satisfactory works of reference we possess, and further emphasises the wisdom of the editor in changing the plan of publication of his Annual to the present form.

---

Atlas and Epitome of Special Pathologic Histology. By Docent Dr. Hermann Durck, Prosector to the Municipal Hospital, Munich. Edited by Ludvig Hektoen, M. D, Professor of Pathology in Rush Medical College, Chicago. Sixty-two colored plates. Philadelphia: W. B. Saunders. 1900. [Price, $3.00.]

This is the latest volume in the Saunders series of Medical Hand-Atlases and it is in keeping with the previous issues. Pathologic histology is a subject of the utmost importance and interest and it is presented here in a manner which is both instructive and pleasing. The plate work is not alone excellent; it is beautifully artistic and, what is more, absolutely correct, so that one studying reproductions can learn quite as much apparently as by viewing the prepared tissues under the microscope.

The main idea which stands out brilliantly throughout the work is to present to the student those changes which are typical of a certain process and the illustrations have been most carefully selected with that end in view.

A study of the plates shows that great and unusual care was exercised by the author in selecting the most typical views of the various processes and in no instance is there the slightest suspicion of schematic representation; on the contrary, so carefully has the work been prepared that the "combinations" of various areas has been studiously and religiously avoided.

In this volume, which is the first of three covering the subject, there are taken up the organs of circulation, the respiratory organs and the digestive organs. The second volume which will issue shortly will complete the subject of special pathologic histology. The third volume will deal with general pathologic histology.

---

### ECKLEY'S PRACTICAL ANATOMY.

NOTE OF CORRECTION.—In the review of Eckley's Practical Anatomy, page 941, July, 1900, it is stated that "the book is a valuable guide to Gerrish's Anatomy" which is an error. As a matter of fact the work is based upon Morris's Anatomy and we regret that our review notice did not contain a definite statement to that effect. We avail ourselves of this opportunity to do all in our power to correct the erroneous statement which we inadvertently published.

